# Lime Formations in the Pulp-Chamber

**Published:** 1893-12

**Authors:** Edward C. Kirk

**Affiliations:** Philadelphia


### LIME FORMATIONS IN THE PULP-CHAMBER.[1]

BY EDWARD C. KIRK, D.D.S., PHILADELPHIA.

LIFE has been defined by Mr. Spencer as "the continuous adjustment of internal relations to external relations," which formula is intended to apply to life in its normal or physiological expression. Aberrations from this standard are what we understand as pathological conditions, and constitute disease. This Ziegler states is a deviation of some of the vital manifestations from the normal: the deviation being conditioned by external influences. It is from the stand-point of this conception of the equilibrium of the vital forces

[1] Read before the Odontological Society of Pennsylvania, October 14, 1893.

in relation to their environment which would seem to best enable us to understand the particular deviation from the normal health of the dental pulp which we are to consider this evening.

The dental pulp functionally considered is the formative sensitive organ of the dentinal structure of the tooth. It is developed in the early weeks of intra-uterine life from the subepithelial connective tissue of the fœtal jaw as the dentinal papilla. This structure in its earlier stages is histologically homogeneous in character, but as development progresses there is formed upon its periphery, about or near the fourth month, a layer of columnar cells, the odontoblasts, which constitute the so-called *membrana eboris.* It is this layer of cells which is immediately concerned in the production of dentine. The deeper portion of the pulp, consisting of embryonal connective-tissue cells, develops a net-work of fibrous connective tissue, with its characteristic bundles of fibres and spindle cells, through which pass the blood-vessels and nerve fibrillæ, and these together constitute the normal tooth-pulp.

The odontoblasts are a modified or specialized form of the connective cells from which they originated. They send out fibrils or rod-like processes towards the enamel, and around these processes is deposited the calcific material giving to the dentine its characteristic tubular structure. The calcific material in question has been called, by Professor Harting, of Utrecht, calcoglobulin. The process of dentine calcification takes place from the periphery towards the centre; hence the area of the pulp becomes gradually diminished through increasing age, during which time the fibrillæ of the odontoblasts elongate, while the cells themselves undergo little or no diminution, simply receding before the advance of the progressing calcification.

It is perhaps not possible to state with precision at what period the process of calcification is normally completed. It has been demonstrated, however, that after a point has been reached where the functional activity of the odontoblasts has naturally ceased, they, having apparently accomplished their normal limit of work, have been again excited through some source of irritation, either local or systemic, to renewed activity, whereby their function has been reawakened and a new deposit of pathological dentine has been thus formed, which is described as secondary dentine. The location, structure, and extent of this secondary dentine formation are no doubt determined by the nature of the irritant which has acted as the exciter. For example, the stimulation may be so localized as to include but a limited area of the membrana eboris, so that the odontoblasts of only that territory will be excited to the deposition of calcoglobulin, while the balance of the pulp will not be affected.

Many instances of this localized formation of secondary dentine are met with in dental practice, and their exciting cause is usually easily ascertained. Thus, in the progress of caries, where the process is so slow that the pulp is not overstimulated through the irritation to the dentinal fibrillæ, we frequently find a deposit of secondary dentine upon that portion of the wall of the pulp-chamber which is being directly approached by the carious process. In fact, as Miller has beautifully shown in his work, "The Micro-Organisms of the Human Mouth," the merest break in the enamel covering is immediately taken cognizance of by the pulp, so that the zone of dentine traversed by the irritated fibrillæ undergoes a certain modification in its structure, which, though not clearly made out as to its character, is still sufficiently marked to constitute a change in texture and refractive properties easily visible under the microscope. When the carious process has proceeded until the pulp is nearly approached, we frequently find a large deposit of secondary dentine which seems to be erected as a barrier to the progress of the carious action. Indeed where the progress of caries has not been sufficiently rapid to overtake the building-up process by the pulp, it is not unusual to find the whole pulp-chamber filled solidly with a deposit of this character.

Sources of irritation other than caries may and do bring about this same deposit. The irritation from thermal changes through metallic fillings frequently induces a local deposit of secondary dentine. Mechanical irritation at the cervical borders of teeth by the abrasive action of brush and powder too vigorously used, ill-fitting clasps upon artificial dentures, erosion of the buccal and labial surfaces of teeth, the mechanical abrasion of their grinding surfaces by attrition of the teeth and their wearing down by mastication, all tend to produce the same condition.

It is necessary at this point to distinguish between certain varieties of calcific changes which take place in the pulp. So far we have concerned ourselves only with those deposits which may in the light of our present knowledge be attributed to excessive functional activity of the odontoblastic layer of the pulp; these are true dentine neoplasms and are always found to be in direct organic union with the walls of the pulp-chamber. Microscopic examinations of these secondary deposits of dentine prove them to be true secretions of the odontoblasts with their fibrillar elements, though often distorted and irregular, directly continuous with those of the normal dentine upon which the secondary deposit is superimposed. But in addition to the secondary deposit just considered, concretions of calcoglobulin are found free in the pulp tissue having no connection with the walls of the pulp-chamber. These concrements present an endless variety in size, shape, number, and histological structure. We find very small, more or less globular nodules, others larger and more irregular in outline, seemingly an aggregation of a number of smaller globular elements united in a mulberry mass, still larger and more irregular masses without any definitely describable shape, or we may find in the same pulp-specimen a variety of masses of many sizes, the whole pulp-tissue being infiltrated with small sandy concrements. Finally, we find nodular growths attached to the walls of the pulp-chamber by a constricted pedicle of the same material. These latter are regarded by Professor Black as true pathological tumors of dentine produced through some perverted action of a localized collection of odontoblasts, and he calls them for that reason odontomes.

The etiology of the formation of free nodular concrements in the pulp tissue is involved in obscurity. While recent investigations have thrown much light upon the structure and histological character of these formations, and a reasonably copious literature has resulted, still it is difficult to find lines of agreement among investigators when the question of the etiology of these formations is at issue. The older writers generally regarded the free calcific growths in the pulp tissue as products of the odontoblastic layer on the hypothesis that they were first developed as secondary dentine in contact with the wall of the pulp-chamber by a pedunculated attachment which was later absorbed, thus setting free the globular end of the growth in the pulp tissue. This view has been definitely proved to be an error. Later researches, notably those of Iszlai, Weil, Hamer, Witzel, Miller, Baume, Black, Bödecker, and many others, have shown that nodular concrements arise in connection with the bundles of fibres and spindle-shaped cells of the connective tissue of the pulp-growth independently of the odontoblasts.

The exact nature of this calcific degeneration of the connective tissue of the pulp is not clearly made out, and it is the various and conflicting views entertained with regard to it that give rise to the confusion respecting the character of these formations as well as to the great variety of names by which they are known. In America they are commonly called "pulp-stones." They have received the names "odontomes," "denticles," "pulp-nodules," "odontheles," etc. One author (Schlenker) has classified them under no less than six different names. The name "odonthele," proposed by Iszlai at the Tenth International Medical Congress at Berlin, is fairly satisfactory; but should, as Miller suggests, be more accurately limited by a prefix which would distinguish these internal pulp formations from the enamel tubercles occurring on the outside of the tooth. The term "endodontheles" would meet the condition more nearly.

The differences in the histological structure of these formations, as seen by different observers, has been largely responsible for the confused state of our knowledge concerning their origin. It is not that the calcific change of connective tissue presents any especially remarkable features, but the resulting product is so variable in its microscopic appearances that it becomes difficult to relate it to the normal processes which result in the formation of the other hard tissues of the body. Thus, nodules will be found presenting a structure closely resembling dentine, while others will be more like bone or cement in character; hence arise conflicting theories as to the method of their formation.

The weight of evidence, so far as the writer from a very imperfect knowledge of the subject may be allowed to judge, seems to show that these formations are the result of certain stimuli local or systemic, perhaps both, whereby the connective tissue of the pulp undergoes a calcific organization characterized by the deposit of calcoglobulin throughout its mass, and that this deposit takes place in relation to the spindle cells of the connective tissue of the pulp in much the same manner as dentine is formed in connection with the odontoblasts. It has not been shown, so far as I have been able to ascertain, whether the spindle cell itself becomes calcified, as it is said to occur in the case of the ameloblast in the formation of enamel, or whether the calcoglobulin is excreted and formed around the fibrillar prolongations of the spindle cells as calco-spherites, which gradually grow by accretion of new material and eventually coalesce with similar formations to form large masses. Hamer, of Utrecht, has shown that the calcification follows the course of the fibres in the bundles of connective-tissue elements; that these latter become embedded in the new formation, giving to it its striated or tubulated appearance, resembling dentine to some extent; as the growth increases small capillary vessels and nerve filaments become included in the mass, which remain as open spaces in the texture of the formation, giving it its relationship in appearance to bone, vaso-dentine, and cement substance.

It will be remembered that the different cellular elements of the pulp tissue had a common origin in the homogeneous cellular structure of the dentinal papilla. The differentiation of these elements is not great morphologically, and it is not unreasonable to suppose that they should present a functional resemblance when they are affected by an irritant which disturbs the vital equilibrium sufficiently to turn the balance towards the pathological side. Over-stimulation of function results in paralysis; the harmonious adaptation of internal relations to external relations has been destroyed, and normal life is no longer possible. In the case of pulp-calcification it does not mean its immediate destruction, for the process may continue over a long period of time; but, given the continuance of the irritation, the result is inevitable, for the pulp-chamber becomes filled with a solid mass and its function is destroyed.

But little is known of the systemic causes which lead to pulp calcification. It is believed that plethoric individuals are more likely to be subject to it than are those of anæmic habit. This view seems probable, inasmuch as the nutritional vigor of such individuals is over-active, and any cause which tends to an increased vascular supply to the pulp favors its calcific degeneration. Of this we have good clinical evidence. So far as age is concerned no class of teeth appears to be exempt. Nodules have been found in non-carious as well as in carious teeth. I observed them in a large number of cases in the first permanent molars of children at the Institution for the Deaf and Dumb in this city some years ago, associated, however, with a pronounced effort at arrestation of the carious process throughout the denture, a result which I have attributed to the greatly improved sanitary and hygienic conditions provided by the institution in comparison with the former condition of the children, which in connection with an abundance of wholesome food had markedly raised the nutritional standard in these cases and, among other expressions, stimulated the pulp to increased functional activity.

The diagnosis of calcific degeneration is extremely difficult where no exposure is present. The most reliable means known to me is the application of cold during the paroxysm of pain supposed to have its origin in this disorder, pain, more or less severe, paroxysmal, and neuralgic in character, being the only well-defined symptom.

The treatment is devitalization and filling of the root-canal. The devitalization of a pulp under those circumstances, as is well known, is a matter often of considerable difficulty. Such pulps show a resistance to the action of arsenious acid which is as persistent as it is remarkable. It can generally be accomplished by repeated trials, but where time is an object it is often preferable to administer a general anæsthetic and remove the contents of the pulp chamber by direct operation. Or the operation may be accomplished under the methyl or ethyl chloride spray. The resistance to the action of arsenic is probably due not to excessive vigor of the pulp, but rather to its diminished vitality with corresponding loss of power to absorb the drug through its altered and diminished vascular system.

The mechanical obstacles presented by nodular concrements in the pulp-chamber are often a great source of difficulty in operating for the removal of a pulp so affected. It can nearly always be accomplished by care and patience with suitable drills and canal reamers.

I have in what I have said merely attempted to outline some of the features of this peculiar condition. There are many interesting phases of it which I trust may have suggested themselves to your minds, and which will be brought out in the further discussion of the subject to-night.

